# Candida Species Isolated From ICU Bloodstream Infections: Molecular Epidemiology, Antifungal Resistance, and Virulence Profiling

**DOI:** 10.7759/cureus.94898

**Published:** 2025-10-19

**Authors:** Raunak Bir, Kuhu Chatterjee, Ankita Rai, Rahul Ranjan, Komal Grover, Yasha Mukim, Anil Pandey, Archana Thakur, Rajiv Gupta, Pooja Pandey

**Affiliations:** 1 Department of Microbiology, Employees' State Insurance Corporation (ESIC) Medical College and Hospital, Faridabad, IND; 2 Department of Microbiology, Institute of Medical Sciences, Varanasi, IND; 3 Department of Microbiology, Amrita School of Medicine, Faridabad, IND; 4 Department of Physiology and Molecular Biology, Employees' State Insurance Corporation (ESIC) Medical College and Hospital, Faridabad, IND

**Keywords:** afst, biofilm, candida spp, fluconazole resistance, hemolysin, icu, mlst, molecular epidemiology, phospholipase

## Abstract

Background and objective: In intensive care units (ICUs), bloodstream infections (BSIs) caused by *Candida* species pose significant clinical problems. This study aims to investigate the species distribution, virulence traits, antifungal resistance profiles, and genetic diversity of *Candida* isolates from patients in Indian ICUs.

Methods: 100 *Candida* isolates were retrieved from the blood samples of ICU patients. The species were identified via molecular and microbiological techniques. Standard Clinical and Laboratory Standards Institute (CLSI)-based procedures were followed for conducting antifungal susceptibility testing. Virulence profiling involved assessing hemolytic activity, the production of hydrolytic enzymes, biofilm formation, and other characteristics linked to pathogenicity. To evaluate relatedness and potential transmission patterns, genetic typing techniques such as multilocus sequence typing (MLST) and sequencing were employed. Additionally, statistical comparisons were performed using the chi-squared test and ANOVA.

Results: Non-*albicans Candida* (NAC) constituted 82% of the isolates. *Candida tropicalis* accounted for 39% of the species distribution, followed by *Candida auris* at 17%, *Candida albicans* at 18%, *Candida parapsilosis *at 10.5%, *Candida glabrata* at 5%, *Candida krusei* at 5%, and other rare species at 5.5%. Around 54% of the isolates showed fluconazole resistance, which was especially high in *C. tropicalis* and *C. auris* (p < 0.0001). About 58% were capable of strong biofilm formation, with the concentration again relying heavily on NAC paddles, which was also shown to be p < 0.0001. Four genetic clades and 29 clustered sequence types were revealed from MLST.

Conclusion: About 90% of resistant strains were non-*albicans Candida* (χ² = 78.57, p < 0.0001), and 54% of isolates of *Candida*, primarily *C. auris *and *C. tropicalis*, showed fluconazole resistance. The minimum inhibitory concentration (MIC) for amphotericin B was low; all isolates were susceptible to echinocandins, and voriconazole remained mainly effective. These results underscore the need for quick species identification and antifungal susceptibility testing, as well as the inadequate efficacy of empirical azole therapy in Indian ICUs. The need of the hour is frequent fungal surveillance, including colonization screening and the identification of prevalent species and susceptibility patterns at the outset to help guide focused empirical treatment. Antifungal drug resistance monitoring assists in stewardship initiatives to reduce the spread of multidrug-resistant *Candida* species, enhancing patient outcomes.

## Introduction

Invasive candidiasis remains a significant cause of illness and mortality among critically ill patients, particularly those in ICUs [1]. *Candida* bloodstream infections (BSIs) are one of the most prevalent healthcare-associated infections in ICUs and are associated with mortality rates as high as 40% in certain groups [2,3]. The increasing incidence of *Candida* BSIs is attributed to many risk factors, including the use of broad-spectrum antibiotics, the presence of central venous catheters, total parenteral nutrition, immunosuppressive therapy, and other invasive medical devices [4].The most often isolated species in cases of candidemia has historically been *Candida* *albicans*. However, non-*albicans Candida* (NAC) species such as *Candida* *tropicalis*, *Candida glabrata*, *Candida parapsilosis*, and *Candida auris* have become more prevalent worldwide in recent times [5]. In addition to having innate or acquired resistance mechanisms, these NAC species have varying antifungal susceptibility profiles, which makes treatment management more challenging and emphasizes the importance of precise species identification and susceptibility testing [6].

Antifungal resistance is becoming a colossal issue in the treatment of candidemia. Increasing numbers of NAC species, particularly *C. glabrata* and *C. tropicalis*, are developing resistance against fluconazole [7]. Echinocandin resistance is less frequent but has been observed and is usually linked to alterations in the FKS1 and FKS2 genes [8]. Mutations in the hotspot regions of the FKS1 and FKS2 genes, which encode the β-1,3-glucan synthase catalytic subunits, are typically associated with echinocandin resistance; these mutations significantly raise minimum inhibitory concentrations (MICs) in comparison to wild-type strains. Multidrug-resistant *C. auris* has frequently resulted in outbreaks; additionally, they exhibit resistance to common antifungal classes (azoles, polyenes, and echinocandins) [9].

Beyond resistance, virulence factors, including adhesins that facilitate attachment to host tissues and medical devices, the creation of biofilms, and the synthesis of hydrolytic enzymes (lipases, phospholipases, and proteases), heighten the pathogenicity of *Candida* [10]. Certain species, like *C. albicans* and *C. tropicalis*, have the ability to switch from yeast to hyphal growth forms, which aids in tissue invasion and immune evasion [11].

Molecular epidemiology, which includes haplotype and polymorphism analysis of internal transcribed spacer (ITS) sequences, offers a more in-depth understanding of the population structure, genetic diversity, and transmission dynamics of the *Candida* species. Recent research has shown clonal spread of resistant *C. auris* lineages both inside and outside of healthcare facilities, emphasizing the necessity of incorporating molecular methods into regular surveillance [12]. *Candida auris* was isolated from 19 units (5.3% of 1,400 cases of candidemia) in a large multicenter intensive care unit (ICU) study that included 27 Indian ICUs. Amplified fragment length polymorphism (AFLP) genotyping showed that the majority of isolates had the same fingerprint, indicating a clonal origin [12].

Due to the constantly changing resistance trends, epidemiology, and variety of virulence factors of* Candida* species, there is a need for continuous surveillance and molecular characterization of clinical isolates. The NAC species, including *C. tropicalis*, *C. parapsilosis*, and *C. glabrata*, have been found to be more common in ICUs in several Indian studies. Nevertheless, the majority of these studies have mostly concentrated on species identification and basic antifungal susceptibility tests, without including information on resistance mechanisms, virulence factors, and molecular typing. Nothing is known about how virulence factors, including adhesion, enzyme synthesis, and biofilm development, contribute to antifungal resistance in India. Furthermore, barely any study has been conducted on the genetic diversity and relatedness of *Candida* isolates that are circulating in ICUs, which leaves significant epidemiological problems unexplored. This lack of knowledge makes it more difficult to identify possible outbreaks or patterns of transmission in critical care units and restricts the creation of focused infection control strategies. Thus, this study intends to comprehensively investigate the genetic diversity, virulence characteristics, antifungal resistance profiles, and species distribution of *Candida* isolates obtained from patients admitted to Indian ICUs. By tackling these undiscovered facets, the study aims to offer a more comprehensive comprehension of the epidemiology and pathogenic potential of BSIs caused by *Candida* in critical care settings. This will aid in tracking any outbreaks or transmission patterns in critical care units. 

## Materials and methods

Study settings

**Figure 1:**
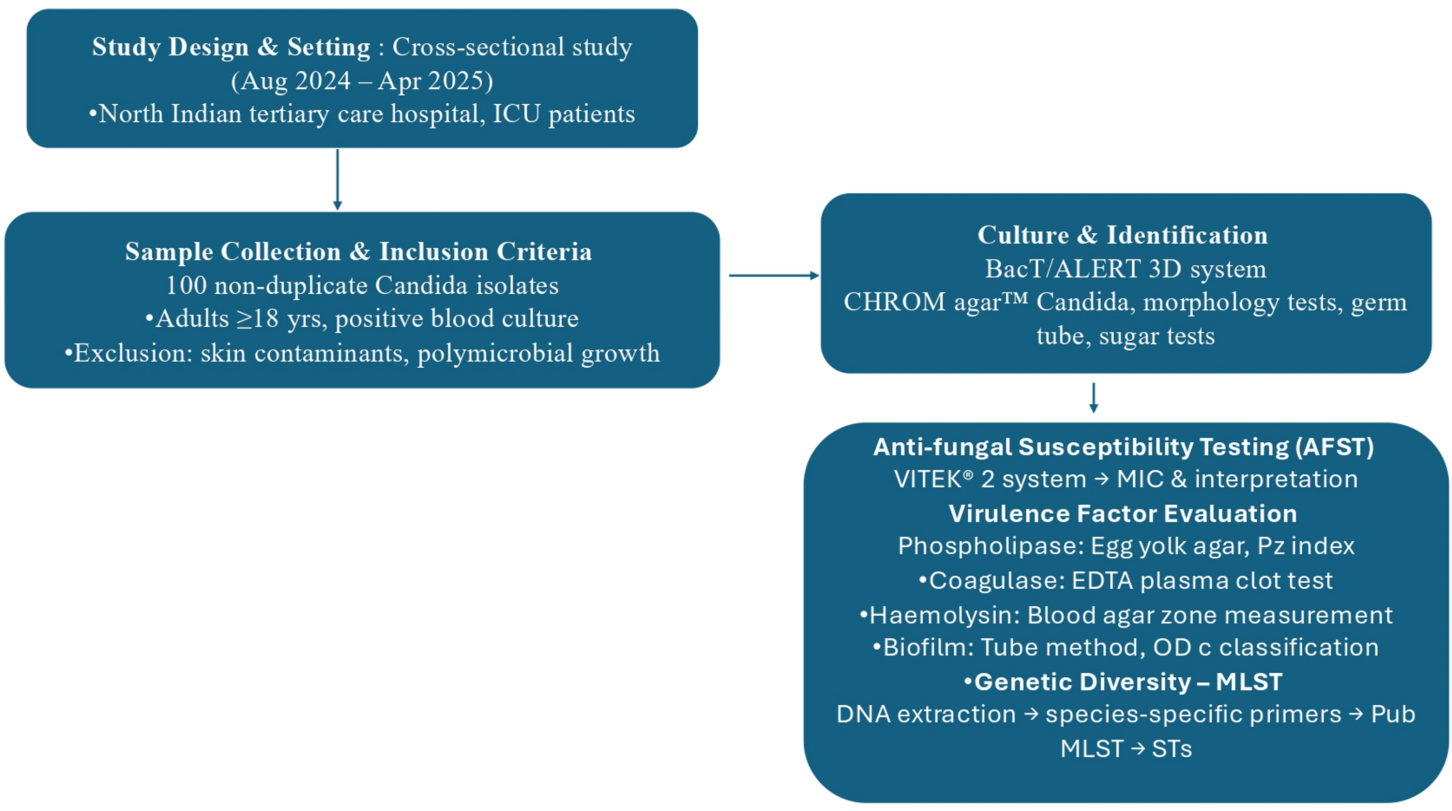
Flow chart depicting the patient recruitment and methods BACT/ALERT®3D (bioMérieux SA, Marcy-l'Étoile, FRA); CHROMagar™ (Kanto Chemical Co. Inc, Chuo-ku, Tokyo, JPN); VITEK® 2 (bioMérieux SA) MIC: Minimum inhibitory concentration, Pz: Phospholipase index, EDTA: Ethylenediaminetetraacetic acid, ODc: Optical density cut-off value, MLST: Multilocus sequence typing, STs: Sequence types

This cross-sectional study was performed from August 2024 to April 2025 at the Department of Microbiology, Employees' State Insurance Corporation (ESIC) Medical College and Hospital (Faridabad, HR, IND), a North Indian tertiary care teaching hospital (Figure 1). The hospital serves a high number of patients and has a high-dependency ICU for the management of critically ill patients across diverse medical and surgical specialties. The Institutional Ethics Committee of ESIC Medical College and Hospital approved the study (approval no. 134 X/11/13/2024-IEC/DHR/102).

A total of 100 non-duplicate consecutive *Candida* isolates were isolated from ICU-admitted patients with BSIs. Adult patients (age ≥18 years) who were admitted to the ICU with one or more positive blood cultures with pure growth of *Candida spp.* and clinical manifestations compatible with BSI were included in the study. Common skin contaminants were excluded from the study, as they can be introduced during blood collection procedures. Cultures showing polymicrobial growth were also excluded from the study. Clinical and microbiological findings relevant to this study were abstracted from hospital records per patient confidentiality and institutional ethical standards. The study's retrospective design resulted in the use of anonymized data taken from pre-existing medical records.

Culture and identification 

The BacT/ALERT 3D system (bioMérieux SA, Marcy-l'Étoile, FRA) was used in a cross-sectional investigation using *Candida* isolates from blood cultures. The finding of *Candida *species in at least one positive blood culture with a pure growth of *Candida spp.* combined with supporting clinical characteristics characterized candidemia. Based on colony color, presumptive species identification of *Candida* isolates was performed by subculturing them on CHROMagar™ Candida (Kanto Chemical Co. Inc., Chuo-ku, Tokyo, JPN) and incubating them at 37°C for 48 hours. Morphological characteristics like chlamydoconidia, blastoconidia, and the development of pseudo hyphae were studied using slide cultures on cornmeal agar supplemented with 1.2% Tween 80. This was complemented by a germ tube test and experiments involving sugar fermentation and sugar assimilation.

Antifungal susceptibility assessments

The VITEK® 2 system (bioMérieux SA) was used for antifungal susceptibility testing (AFST) of* Candida spp.* The test was performed per manufacturer instructions. The antifungal susceptibility test-yeast (AST-YS0 cards used provide MIC values and interpret results based on established breakpoints [13]. 

Evaluation of virulence factors 

The virulence potential of *Candida* isolates was assessed by measuring biofilm development, coagulase synthesis, phospholipase, and hemolysin activities in accordance with accepted procedures. Phospholipase activity was evaluated on egg yolk agar, which consisted of 10% sterile egg yolk supernatant, sodium chloride (NaCl), calcium chloride (CaCl₂), and Sabouraud dextrose agar [14]. A 5 μL inoculum (about 108 cells/mL) was applied, the plates were allowed to dry at room temperature, and they were incubated at 37°C for 48 hours. The phospholipase index (Pz), which is the ratio of the colony diameter to the total diameter of the colony plus the precipitation zone, was used to measure the phospholipase activity. Enzymatic activity was indicated by Pz < 1. 

The manufacturing of coagulase was determined by mixing 0.1 mL of an overnight *Candida* culture with 500 μL of ethylenediaminetetraacetic acid (EDTA) plasma and incubating the mixture for four hours at 35°C. The result was deemed positive if an insoluble clot that could withstand mild agitation was present [15]. Tubes were re-incubated and retested at 24 hours if they were negative at four hours. Staphylococcus aureus ATCC 25923 and *Staphylococcus epidermidis* ATCC 14990 were utilized as positive and negative controls, respectively. 

Using Sabouraud dextrose agar enriched with 3% glucose and 7 mL of sheep blood per 100 mL of medium, the hemolytic activity was determined [16]. After applying a 10 μL inoculum (~108 cells/mL), the sample was incubated for 48 hours at 37°C. Hemolytic activity was calculated as the colony diameter divided by the hemolysis zone diameter. Positive controls were *Streptococcus sanguis* and *Streptococcus pyogenes* for alpha and beta hemolysis, respectively. 

Biofilm formation was determined by the tube method [17]. The colonies were kept in saline at 37°C for 24 hours. Post which, 0.5 mL of culture was inoculated into polystyrene tubes filled with Sabouraud dextrose broth with 8% glucose and incubated for 48 hours at 37°C without shaking. Tubes were washed gently, stained with 2% crystal violet for 10 minutes, and biofilm positivity was defined by visible film formation on the tube walls and bottoms. The *S. epidermidis* ATCC 35984 and *C. albicans* ATCC 10231 were used as positive and negative controls, respectively. The optical density cut-off value (ODc) was calculated for quantitative validation by measuring the OD of the negative control (sterile broth) and calculating ODc as the average OD of the negative control plus three standard deviations. Non/weak biofilm producers had OD ≤ ODc, moderate biofilm producers had ODc < OD ≤ 2×ODc, while strong biofilm producers had OD > 2×ODc.

Multilocus sequence typing (MLST)

To evaluate the genetic diversity of *Candida* isolates, MLST was carried out using species-specific procedures where standardized schemes were available. Following the manufacturer's instructions, genomic DNA was extracted from pure yeast colonies cultured on Sabouraud dextrose agar using a Qiagen DNA extraction kit (Qiagen, Hilden, DEU) [18].

Species-specific primer sets to amplify housekeeping genes

According to the PubMLST scheme, for *C. albicans* AAT1a, ACC1, ADP1, MPIb, SYA1, VPS13, and ZWF1b were used. For *C. glabrata*, FKS, LEU2, NMT1, TRP1, UGP1, and URA3 were used. The ICL1, MDR1, SAPT2, SAPT4, XYR1, ZWF1 for *C. tropicalis*. For *C. parapsilosis, *SADH, SYA1, and additional species-specific loci were used. For* C. auris*, there was no defined MLST scheme; thus, the isolates were not typed using MLST. Instead, molecular typing was done using whole-genome sequencing analysis [19].

Assignment of alleles and determination of sequence type (ST)

Bidirectional sequences were matched against reference alleles in the PubMLST database after being assembled and modified using MEGA X. Every distinct allele combination was given an ST, and allele numbers were allocated according to accurate sequence matches. For curation, novel alleles or STs were added to the database [20].

Allelic profiles (STs) were combined into a table in an XLS format that was compatible with the analysis program. BioNumerics v7.6 (Applied Maths, Sint-Martens-Latem, BEL), a platform developed for MLST data visualization, was used to create a minimum spanning tree (MST). The number of different loci between each ST was used by the software to compute pairwise distances [21].

Statistical analysis 

Data were analyzed using SPSS Statistics version 20.0 (IBM Corp., Armonk, NY, USA). Categorical variables were compared using the chi-square (χ²) test or Fisher’s exact test where appropriate. Statistical significance was defined as p < 0.05 for two-sided tests.

## Results

Species distribution among ICU isolates

A total of 100 *Candida* isolates were obtained from blood samples of ICU patients during the study period. The distribution revealed a predominance of NAC species, accounting for 82% of the isolates. The most frequently identified species was *C. tropicalis* (39%), followed by *C. auris *(17%) and *C. albicans* (18%). Other species included *C. parapsilosis* (11%), *C. glabrata* (5%), *Candida krusei *(5%), and a minority (6%) of other uncommon *Candida spp*. These findings reflect the increasing shift towards NAC species, particularly *C. tropicalis *and *C. auris*, in Indian intensive care settings.

Virulence factor expression

Biofilm Formation

Using the tube method, biofilm production was mainly evaluated and visually scored utilizing crystal violet staining. Depending on the degree of adherent stained film on the tube walls and bottoms, isolates were classified as strong, moderate, or weak biofilm producers. To facilitate interspecies comparison, a quantitative evaluation was also carried out utilizing a microtiter plate assay based on crystal violet. Optical density measurements revealed that 58% of isolates formed strong biofilms (OD > 0.6), 25% formed moderate biofilms (OD 0.3-0.6), and 17% formed weak biofilms (OD < 0.3). Most often, isolates of *C. tropicalis *and *C. auris* showed strong biofilm production, whereas isolates of* C. albicans* mostly showed a moderate capacity to produce biofilms (Table 1). The ANOVA showed that OD values varied significantly between species (F = 69.74, p < 0.0001).

**Table 1:** Stratification of biofilm formation capacity among Candida isolates in the study OD: Optical density

Biofilm category	OD range	No. of isolates (n)	Percentage of isolates	Predominant species
Strong	> 0.6	58	58%	*C. tropicalis*, *C. auris*
Moderate	0.3–0.6	25	25%	C. albicans
Weak	< 0.3	17	17%	Mixed

Hydrolytic Enzyme Production

Phospholipase activity was detected in 50% of isolates, particularly among *C. tropicalis* and *C. auris*. These results support their role in enhanced pathogenicity and tissue invasion (Table 2).

**Table 2:** Distribution of phospholipase activity and anti-fungal resistance

Species	Phospholipase positive (%)	Fluconazole resistance (%)	Voriconazole susceptibility	Amphotericin B	Echinocandins
C. tropicalis	High	High	Mostly susceptible	Susceptible	Susceptible
C. auris	High	High	Reduced susceptibility	Susceptible	Susceptible
C. albicans	Moderate	Low	Susceptible	Susceptible	Susceptible
C. glabrata	Low	High	Variable	Susceptible	Susceptible
C. krusei	Low	Intrinsic	Intrinsic	Susceptible	Susceptible
C. parapsilosis	Moderate	Low	Susceptible	Susceptible	Susceptible

Anti-fungal susceptibility patterns

Of the isolates, 54% had resistance to fluconazole. Particularly noticeable resistance was seen in *C. tropicalis*, *C. glabrata*, and *C. auris*. While isolates resistant to fluconazole frequently showed decreased susceptibility, most isolates' MICs for voriconazole stayed within susceptible limits. The majority of isolates remained sensitive to amphotericin B and had low MICs. There was no resistance to echinocandins (micafungin and caspofungin), and both drugs maintained their activity across species (Table 2). 

Isolates were categorized as resistant if their fluconazole MICs were higher than 32 µg/mL. Around 90% of them were NAC species, underscoring the difficulty of empirical azole therapy in Indian ICUs. Notably, 54% of isolates in this cohort were resistant to fluconazole, with resistance rates significantly varying by species (χ² = 78.57, p < 0.0001). Both C. auris and C. tropicalis accounted for most of the resistant strains, consistent with prior Indian multi-center studies reporting azole resistance in 11.8% to 17% of isolates. This resistance undermines the utility of fluconazole as empiric therapy and necessitates early species-level identification and susceptibility testing.

Genetic diversity by MLST

Bloodstream isolates from six clinically significant species of *Candida *were subjected to MLST. The evaluation of intra-species diversity and possible clonal linkages was made possible by the analysis of species-specific loci to identify allele profiles and assign STs. The isolates of *C. albicans *were examined for seven housekeeping genes: AAT1a, ACC1, ADP1, MP1b, SYA1, VPS13, and ZWF1b. Distinct STs were assigned since each isolate displayed a different combination of alleles across the loci. With no obvious signs of clonal clustering, the observed variety points to several distinct strains circulating throughout the patient group. Four loci were used in the MLST of C. auris: TS1/ITS2, D1/D2, RPB1, and RPB2. The probability of transmission from a common source is supported by this high level of allelic conservation, which is in line with the known clonal epidemiology of *C. auris* in nosocomial settings. We used six loci, FKS, LEU2, NMT1, TRP1, UGP1, and URA3, to examine isolates of *C. glabrata*. Clonality is indicated in this data, which also raises the possibility of nosocomial transmission or acquisition from a common environmental reservoir. The examined isolates showed no signs of diversification.

The HIS3, LEU2, NMT1, TRP1, ADE2, and LUS2D were among the six loci employed in the C. krusei MLST. Allelic changes in several loci were used to distinguish these STs from one another, indicating a moderate level of genetic diversity among the isolates. This variation could be the result of underlying evolutionary divergence or independent acquisition events. Because a validated and comprehensive MLST strategy for the sequenced loci was not available, MLST was not conducted for C. parapsilosis isolates. For C. tropicalis, six loci were examined: ICL1, MDR1, SAPT2, SAPT4, XYR1, and ZWF1a. A single locus variant (SLV) link was seen; it may be due to microevolutionary events that may have occurred inside the healthcare context. This indicates a tight genetic link and likely recent divergence from a common ancestor (Table 3).

**Table 3:** Details of genetic diversity by MLST among Candida isolates MLST: Multilocus sequence typing, SLV: Single locus variant

Species	Loci analyzed	Clonality observed
C. albicans	AAT1a, ACC1, ADP1, MP1b, SYA1, VPS13, ZWF1b	No
C. auris	TS1/ITS2, D1/D2, RPB1, RPB2	Yes
C. glabrata	FKS, LEU2, NMT1, TRP1, UGP1, URA3	Yes
C. krusei	HIS3, LEU2, NMT1, TRP1, ADE2, LUS2D	No
C. parapsilosis	Not typed (no validated scheme)	—
C. tropicalis	ICL1, MDR1, SAPT2, SAPT4, XYR1, ZWF1a	Partial (SLV)

**Figure 2:**
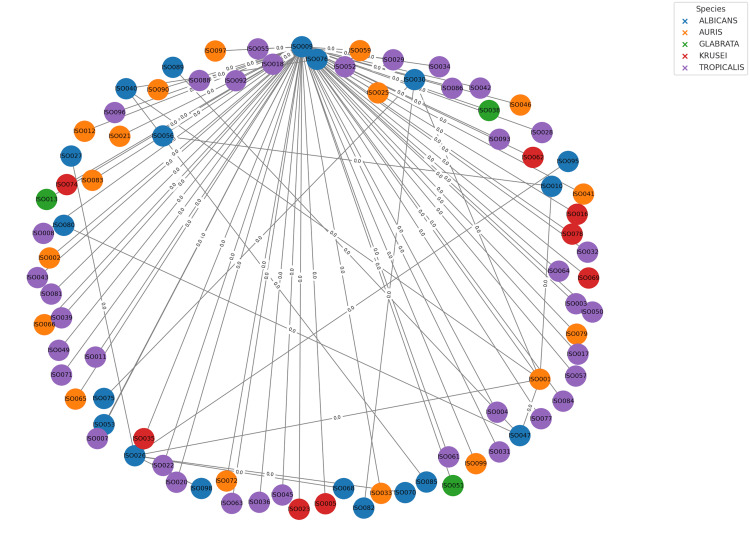
The MST illustrating genetic relationships among Candida isolates constructed using BioNumerics version 7.6 Labeled with its unique identifier, each node represents a single isolate. *C. albicans* (blue), *C. auris *(orange), *C. glabrata* (green), *C. krusei* (red), and *C. tropicalis* (purple) are the species-specific color-coded nodes. Allelic differences are shown by edge weights across nodes, which are proportional to the number of loci that differ between sequence types. Dispersed nodes exhibit allelic variety, while closely related isolates group together, demonstrating genetic proximity and potential clonal linkages. MST: Minimum spanning tree

The MLST analysis was employed to determine the genetic relatedness of the *Candida* isolates using allelic profiles. GrapeTree, a visualization program for showing relationships between sequence types, was employed [22]. The MST illustrating genetic relationships among *Candida* isolates is documented in Figure 2.

## Discussion

The prevalence of NAC species, especially *C. tropicalis* and *C. auris*, in ICU-acquired candidemia highlights the urgent need for revised clinical procedures. This study demonstrates and reaffirms a concerning shift toward NAC species in Indian ICU settings, particularly *C. tropicalis* and *C. auris*. These species display significant azole resistance and potent virulence, including biofilm formation and hydrolytic enzyme activity. The resistance patterns and genetic diversity highlight the need for hospital-specific treatment guidelines and infection control policies.

Our findings are consistent with prior reports by Chakrabarti et al. and Mathur et al., which have noted *C. tropicalis* as a leading pathogen in India with rising resistance trends. These findings align with national surveillance data, where NACs have overtaken* C. albicans* as the primary etiologic agents of candidemia, particularly in tropical regions such as India and Southeast Asia. The detection of C. auris in 17% of cases is particularly concerning, given its potential for nosocomial transmission and pan-resistance [23]. Strong biofilm formation was significantly more frequent in *C. tropicalis* and *C. auris*, as shown by OD values (F = 69.74, p < 0.0001). Biofilms contribute to antifungal resistance and device-associated infections, especially in ICU patients with central lines or catheters. 

Genetic heterogeneity and epidemiological implications

The significant variations in clonality and population structure were found in the MLST analysis of bloodstream isolates from six clinically significant species of *Candida*, which have substantial ramifications for epidemiological monitoring and infection control. As each isolate of *C. albicans *represented a different sequence type, the isolates showed substantial allelic diversity across seven housekeeping genes, indicating numerous independent acquisition events rather than nosocomial transmission [24,25]. In contrast, *C. auris* isolates showed strong allelic conservation across four loci, consistent with clonality and likely hospital transmission from a common source, highlighting the need for stringent infection control measures. This is consistent with other research that identified *C. auris* as a clonal species that is frequently linked to hospital outbreaks and ongoing colonization of healthcare settings [26,27]. Comparably, isolates of *C. glabrata* showed little allelic variation, which might be a result of recent transmission events or the dominance of a clonal lineage under the influence of antifungal use-induced selection. Numerous studies have linked hospital transmission and selective pressures from antifungal medications, especially echinocandins, to clonality in *C. glabrata* [28,29]. The *C. tropicalis* isolates showed closely similar sequence types with SLVs, suggesting microevolution in response to host and surroundings, whereas* C. krusei* showed moderate genetic diversity, suggesting various infection sources [30,31]. Because there was no approved scheme, MLST could not be carried out for *C. parapsilosis*. Overall, these results highlight species-specific epidemiological patterns; genetically diverse species (*C. albicans* and *C. krusei*) reflect multiple independent introductions, guiding targeted surveillance and infection control strategies, while highly clonal species (*C. auris* and *C. glabrata*) may be the cause of nosocomial outbreaks.

Clinical relevance and public health impact

Our findings align with the conclusions of the landmark Indian study by Chakrabarti et al., which identified *C. tropicalis* as the predominant NAC species in Indian ICUs, characterized by increasing rates of antifungal resistance and attributable mortality. The identification of *C. auris* in 17% of isolates is particularly alarming given its association with clonal outbreaks, environmental persistence, and pan-resistance risks.

Limitations

This is a single-center study, which may not accurately reflect trends across the country. The absence of environmental sampling limited our understanding of resistance mechanisms and transmission pathways. Furthermore, in-depth clinical outcome associations were not evaluated, and MLST analysis was limited to species with known schemes.

## Conclusions

An important epidemiological change has occurred with the emergence of NAC species as a major source of BSI in ICUs. The high prevalence of azole resistance among NAC in Indian ICUs is highlighted by the fact that 90% of resistant isolates were of the NAC species. Fluconazole resistance was present in 54% of the *Candida *isolates in this ICU cohort, with resistance being most significantly linked to *C. auris*, *C. tropicalis*, and *C. glabrata*. Voriconazole MICs stayed mostly within susceptible ranges, and all isolates continued to be sensitive to echinocandins (micafungin and caspofungin), despite the prevalence of fluconazole resistance. The activity of amphotericin B was also preserved, and the majority of isolates showed low MICs. Fluconazole resistance rates were highly species-specific, highlighting the limited effectiveness of empirical azole therapy and the necessity of early species-level identification and susceptibility testing in ICUs.

The frequent occurrence of multidrug-resistant biofilm-forming strains underscores the importance of antifungal susceptibility testing, timely species-level identification, and the consideration of echinocandins in high-risk patients. Enhancing molecular typing and surveillance systems is essential for tracking evolving resistance and preventing outbreaks. Improved infection control measures, hospital-specific antifungal stewardship, and continuous monitoring of emerging lineages are critical to addressing this growing challenge. Therefore, customized diagnostic, therapeutic, and preventive strategies are essential to reducing the clinical and public health risks. 
